# Antibacterial Efficacy of Lytic Bacteriophages against Antibiotic-Resistant *Klebsiella* Species

**DOI:** 10.1100/tsw.2011.114

**Published:** 2011-07-07

**Authors:** M. Khajeh Karamoddini, B. S. Fazli-Bazzaz, F. Emamipour, M. Sabouri Ghannad, A. R. Jahanshahi, N. Saed, A. Sahebkar

**Affiliations:** ^1^ Department of Microbiology, Ghaem Medical Center, Mashhad University of Medical Sciences, Mashhad, Iran; ^2^ Biotechnology Research Center and School of Pharmacy, Mashhad University of Medical Sciences, Mashhad, Iran; ^3^ Department of Microbiology, School of Medicine, Hamadan University of Medical Sciences, Hamadan, Iran; ^4^ Cardiovascular Research Center, Avicenna Research Institute, Mashhad University of Medical Sciences, Mashhad, Iran

**Keywords:** bacteriophage, Klebsiella, antibiotic resistance

## Abstract

Bacterial resistance to antibiotics is a leading and highly prevalent problem in the treatment of infectious diseases. Bacteriophages (phages) appear to be effective and safe alternatives for the treatment of resistant infections because of their specificity for bacterial species and lack of infectivity in eukaryotic cells. The present study aimed to isolate bacteriophages against *Klebsiella* spp. and evaluate their efficacy against antibiotic-resistant species. Seventy-two antibiotic-resistant *Klebsiella* spp. were isolated from samples of patients who referred to the Ghaem Hospital (Mashhad, Iran). Lytic bacteriophages against *Klebsiella* spp. were isolated from wastewater of the septic tank of the same hospital. Bactericidal activity of phages against resistant *Klebsiella* spp. was tested in both liquid (tube method; after 1 and 24 h of incubation) and solid (double-layer agar plate method; after 24 h of incubation) phases. In each method, three different concentrations of bacteriophages (low: <10^4^ PFU/mL, medium: 10^4^–10^7^ PFU/mL, and high: > 10^7^ PFU/mL) were used. Bacteriophages showed promising bactericidal activity at all assessed concentrations, regardless of the test method and duration of incubation. Overall, bactericidal effects were augmented at higher concentrations. In the tube method, higher activity was observed after 24 h of incubation compared to the 1-h incubation. The bactericidal effects were also higher in the tube method compared to the double-layer agar plate method after 24 h of incubation. The findings of the present study suggest that bacteriophages possess effective bactericidal activity against resistant *Klebsiella* spp. These bactericidal activities are influenced by phage concentration, duration of incubation, and test method.

## INTRODUCTION

Bacteriophages (also called phages) are reported to be the most abundant organisms on earth[1] and are ubiquitous in nature. More than 5000 classified bacteriophages are known[2]. Phages are easily identified in water sources, sewage, and soil[3].

Based on the replication type, phages are classified as either lytic or lysogenic. A lytic phage replicates in the bacterial host and destroys its host in a process, but a lysogenic phage inserts itself into the genome of its bacterial host and establishes a stable position in the infected bacterium[4]. Lysogenic phages may transfer genes that express toxin proteins or pathogen factors among bacterial species[5].

After discovery, phages were the target of multiple research for the treatment of bacterial diseases, such as dysentery[6]. In the 1920s and 1930s, Lilly and Squib worked on the preparation of phages for the treatment of *Staphylococcus* infections. However, a number of factors, including the discovery of antibiotics, caused less attention to be focused on the medical application of phages.

In spite of the great progress that has been made in the field of antimicrobial therapy, the appearance and spread of drug-resistant bacteria has caused a serious challenge in recent decades. As an example, the prevalence of resistant nosocomial infections is increasing at an alarming rate and their elimination is very difficult. This could be secondary to the wide use of antibiotics, as well as application of therapeutic measures that weaken the immune system and make subjects more susceptible to nosocomial infections. Phage therapy could be an effective alternative approach for the control of these infections, as several studies have shown their efficacy against both Gram-positive and Gram-negative bacteria[7,8,9,10,11,12]. In addition, phages might be associated with less side effects compared to antibiotics, which is due to the lack of phage receptors on eukaryotic cells[13,14,15,16].

The purpose of the present study was to isolate and enrich lytic bacteriophages against *Klebsiella* spp. and evaluate their antibacterial efficacy against antibiotic-resistant species. The impact of phage concentration, incubation duration, and method of culture (tube vs. plate) on the bactericidal effect was also investigated.

## MATERIALS AND METHODS

### Isolation of *Klebsiella* spp.

Different samples, mainly from urine, vaginal smears, blood, wounds and their secretions, and burn lesions, were collected from patients referring to the Ghaem Hospital (Mashhad, Iran) during a course of about 1.5 years between November 2001 and March 2003. Samples were cultured on general (simple blood agar; supporting the growth of most microorganisms) as well as specific (MacConkey agar, desoxycholate agar, or eosin methylene blue agar; supporting the growth of Gram-negative bacteria) culture media. Culture media plates were incubated at 37°C for 24 h. To confirm the isolation of *Klebsiella* spp., Gram staining and multiple biochemical tests were performed, including glucose and lactose fermentation (Kligler iron agar medium), citrate utilization (Simmons citrate agar medium), urea (urea agar medium), hydrogen sulfide production, indole formation and motility (sulfide-indole-motility [SIM] agar medium; Kligler iron agar medium), and malonate utilization (malonate agar medium) tests.

### Determination of *Klebsiella* spp. Sensitivity to Antibiotics

Mueller-Hinton agar medium was used to culture the appropriate bacteria. Colonies were first suspended in 5 mL of tripticase soy broth and kept at 37°C for several hours until the turbidity of the suspension changed, similar to that of barium sulfate solution in the 0.5 McFarland standard tube (the standard tube was shaken vigorously before usage). A sterile swab was stirred in the above suspension and the sample was cultured on Mueller-Hinton agar medium. Antibiotic disks were placed at a 15-mm distance from the edge of the plate. Different disks were 24 mm from the center of each nearest disk. Following a 24-h incubation at 37°C, the growth inhibition zone was measured and compared with tables provided by the National Committee for Clinical Laboratory Standards (NCCLS). The results of sensitivity were reported as sensitive, resistant, or intermediate. Antibiotics that were evaluated included ampicillin, amoxicillin, amikacin, cephalexin, chloramphenicol, nitrofurantoin (for urine samples), gentamicin, kanamycin, nalidixic acid (for urine samples), rifampin, streptomycin, tetracycline, doxycycline, tobramycin, and sulfamethoxazole. Smooth agar containing glycerin was used to keep resistant *Klebsiella* colonies at -20°C as follows: four to five colonies were transferred to 20 mL of triple soy broth. After 4 h of incubation at 37°C, the tube containing tryptone soy broth was centrifuged at 2500 rpm. Then, 0.5 mL of the above-cultured bacteria was transferred to a Pyrex® test tube containing 3 mL of 3% Mueller-Hinton. Test tubes were incubated at 37°C for 4–6 h in order to accelerate bacterial growth. Following that, 0.5 mL of sterile glycerin was added to test tubes and tubes were transferred to -20°C.

## Isolation, Enrichment, Titration, and Bacteriophages

Bacteriophages utilized in this study were isolated from wastewater of the septic tank in Ghaem Hospital that had been filter sterilized. To the aforementioned wastewater (45 mL), concentrated nutrient broth medium (5 mL) and 4-h antibiotic-resistant *Klebsiella* culture (5 mL) were added. Also added was 1% (v/w) $MgSo_4$ to provide optimum attachment of bacteriophage to bacteria. The mixture was then gently shaken and kept at 37°C for 24 h. Afterwards, chloroform was added (3 mL) and the mixture was shaken for 15 min. After being kept at room temperature for 2 h, the mixture was centrifuged (30 min, 3500 rpm) and the supernatant carefully isolated. For phage enrichment, the obtained supernatant was mixed with nutrient broth (10 mL) and 4-h *Klebsiella* culture (2 mL). The mixture was then processed as described above. Phage suspension was maintained in the nutrient broth at 4°C in a dark place using sterile and sealed glass containers.

For the titration of phages, enriched samples were diluted by 10X in tubes containing 9 mL of tryptone broth. Then, 100 µL of each diluted sample was transferred to tubes containing 3 mL of soft agar. Afterwards, 4-h *Klebsiella* culture (1 mL) was added to each tube. Tubes were then shaken and their contents rapidly transferred to plates containing tryptone agar medium. The plates were incubated at 37°C for 24 h. Plates containing 30–300 plaques were used to calculate the number of phages in the primary solution using the following formula:

$$\textit{Number of phages} = \textit{Number of plaques} \times \textit{dilution titer} \times \textit{volume of media}$$

## Evaluation of Antibacterial Activity

The antibacterial effects of phages against antibiotic-resistant *Klebsiella* spp. were tested by the tube method and the double-layer agar plate method at two time points: after 1 h (for the tube method) and 24 h (for both tube and plate methods) of incubation at 37°C. In each method, three different concentrations of phages were tested: low ($<10^4$ PFU/mL), medium ($10^4$–$10^7$ PFU/mL), and high ($>10^7$ PFU/ mL). According to the intensity of growth inhibition, the results were reported as +++ (75–100% reduction of bacteria compared to control), ++ (50–75% reduction of bacteria compared to control), + (25–50% reduction of bacteria compared to control), and - (<25% reduction of bacteria compared to control).

## Statistical Analysis

All comparisons were performed using Fisher’s exact test. A two-sided $p$ value of $<0.05$ was considered to be statistically significant.

## RESULTS

Out of the total samples that were collected during the course of the study (a period of approximately 1.5 years), 72 antibiotic-resistant *Klebsiella* spp. were isolated. Most of these species were isolated from urine, wounds, and burn lesion samples (Fig. 1). The antibiotic resistance of these species was tested using several antibiotics. Ampicillin and amoxicillin had the highest rate of resistance (Table 1). Among the isolated, resistant *Klebsiella* spp., *K. pneumoniae* was the most prevalent (n = 67). *K. oxytoca* (n = 3), *K. ozaenae* (n = 1), and *K. rhinoscleromatis* (n = 1) constituted only a small fraction of isolated species.

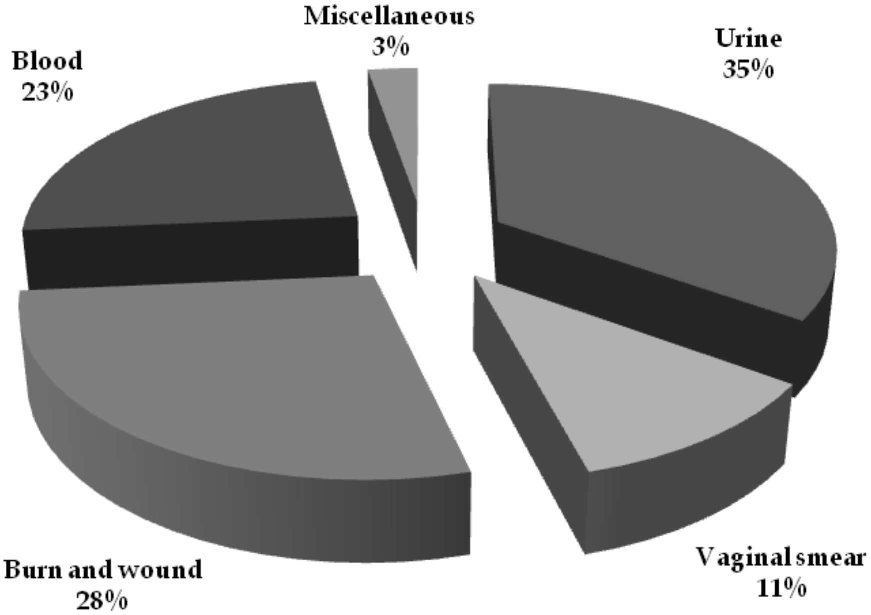

**FIGURE 1.** Distribution of isolated *Klebsiella* spp. among different specimens.

**TABLE 1**
**Antibacterial Activity of Different Phage Concentrations against Resistant *Klebsiella* spp. after 1 and 24 h of Incubation in the Tube Method**

| Concentration | Incubation Time (h) | - | + | ++ | +++ |
|---|---|---|---|---|---|
| Low (<$10^4$ PFU/mL) | 1 | 0 (0) | 48 (66.7) | 24 (33.3) | 0 (0) |
| | 24 | 0 (0) | 0 (0) | 36 (50) | 36 (50) |
| Medium ($10^4$–$10^7$ PFU/mL) | 1 | 0 (0) | 19 (26.4) | 52 (72.2) | 1 (1.4) |
| | 24 | 0 (0) | 0 (0) | 15 (20.8) | 57 (79.2) |
| High (>$10^7$ PFU/mL) | 1 | 0 (0) | 0 (0) | 21 (29.2) | 51 (70.8) |
| | 24 | 0 (0) | 0 (0) | 10 (13.9) | 62 (86.1) |
| Overall concentration effect | 1 | $p < 0.001$ | | | |
| | 24 | $p < 0.001$ | | | |

Values are expressed as number (%). Comparisons were made using Fisher's exact test.

In the tube method, different concentrations (low, medium, and high) of phages were evaluated for their inhibitory effect against the growth of isolated, resistant *Klebsiella* spp. after 1 and 24 h of incubation at 37°C. The results indicated that in both time points, all three assessed concentrations had antibacterial effects without even one strain being unaffected by phage treatment. There was a marked increase in the antibacterial effects after 24 h compared to 1 h of incubation, and this was observed for all three assessed phage concentrations. There was also a positive association between phage concentration and observed antibacterial effects at both assessed time points. This effect of concentration was found to be of high statistical significance when comparing the antibacterial effects of low concentration to those of medium ($p < 0.001$) and high ($p < 0.001$) concentrations. However, while there was a significant concentration effect at the 1-h incubation time point between medium and high phage concentrations ($p < 0.001$), no significant difference was observed after 24 h of incubation ($p > 0.05$) (Table 2).

**TABLE 2**
**Antibacterial Activity of Different Phage Concentrations against Resistant *Klebsiella* sp. after 24 h of Incubation in the Double-Layer Agar Plate Method**

| Concentration | Incubation Time (h) | - | + | ++ | +++ |
|---|---|---|---|---|---|
| Low (<$10^4$ PFU/mL) | 1 | 0 (0) | 48 (66.7) | 24 (33.3) | 0 (0) |
| Medium ($10^4$–$10^7$ PFU/mL) | 1 | 0 (0) | 19 (26.4) | 52 (72.2) | 1 (1.4) |
| High (>$10^7$ PFU/mL) | 1 | 0 (0) | 0 (0) | 21 (29.2) | 51 (70.8) |
| Overall concentration effect | | $p < 0.001$ | | | |

Values are expressed as number (%). Comparisons were made using Fisher's exact test.

Aside from the tube test, the antibacterial activity of different phage concentrations was tested using the double-layer agar plate method after 24 h of incubation at 37°C. The results further confirmed the antibacterial effects of phage preparations and the increased activity at medium ($p < 0.001$) and high ($p < 0.001$) compared to low concentrations. The effects of medium and high concentrations were again comparable ($p > 0.05$) (Table 3). Of note, higher antibacterial efficacy was observed in the tube method compared to that of plate method (after 24 h) at low ($p < 0.001$) and medium ($p < 0.001$), but not high, phage concentrations ($p = 0.042$).

## DISCUSSION

The most obvious result to emerge from the present study was the promising antibacterial effects of phages against resistant *Klebsiella* spp. at all assessed (low, medium, and high) concentrations. The results also indicated that bactericidal effects of phages are augmented with increasing concentration and time of incubation. In addition, the double-layer agar plate method was associated with higher bactericidal effects compared to the tube method.

Bactericidal effects of phages at low concentrations are due to their self-replication property. At low concentrations, the number of phages is exponentially increased in the presence of bacterial host[13]. Therefore, only a small number of phages suffice for the elimination of bacterial infection. Along with the decline in bacterial population, the number of phages is also decreased, leading to the gradual elimination of phages from the environment (self-limiting property)[13,17]. Notably, upon reinfection with the same type of bacteria, phages will again start to replicate[18]. This characteristic of phages allows their possible application for preventive purposes.

**TABLE 3**
**Prevalence of Antibiotic Resistance among Isolated *Klebsiella* spp.**

| Antibiotic | Resistant (%) | Intermediate (%) | Sensitive (%) |
|---|---|---|---|
| Ampicillin | 80 | 1 | 19 |
| Amoxicillin | 77 | 3 | 20 |
| Amikacin | 21 | 1 | 78 |
| Streptomycin | 20 | 0 | 80 |
| Tetracycline | 28 | 2 | 70 |
| Tobramycin | 18 | 1 | 81 |
| Gentamicin | 19 | 1 | 80 |
| Doxycycline | 24 | 1 | 75 |
| Rifampin | 69 | 28 | 3/3 |
| Cephalexin | 22 | 2 | 78 |
| Sulfamethoxazole | 25 | 0 | 75 |
| Kanamycin | 28 | 1 | 71 |
| Chloramphenicol | 25 | 0 | 75 |
| Nalidixic acid | 28 | 1 | 81 |
| Nitrofurantoin | 19 | 0 | 81 |

As confirmed by the present results, phages have bactericidal properties at both low and high concentrations. These bactericidal effects are exerted through active (at low concentrations) or passive (at high concentrations) mechanisms. In the active mechanism, the number of phages is lower than the bacterial host, resulting in the proliferation of phages in the bacteria. In this mechanism, the bactericidal effects are mainly due to the lytic activity of daughter phages. In contrast, the passive mechanism occurs when the primary infecting phages are in excess compared to the bacterial host. Therefore, most of the lytic effects would be exerted by the primary phages[19,20,21,22]. Passive therapy has been demonstrated to have several advantages over active therapy, including simpler dynamics and lower occurrence of bacterial resistance. In addition, as phage replication is not a requisite for passive therapy, this mechanism does not appear to be adversely influenced by restriction modifications, abortive infections, or interference (in case of multiple infections)[23].

Phages possess some unique properties that make them promising candidates for the treatment of bacterial infections. First, they need to bind to specific surface receptors in order to enter the bacteria and exert their effects. Hence, their bactericidal effects would be specific. Second, since eukaryotic cells lack phage receptors, phage preparations appear be harmless to human, animal, and plant cells[13,14,15,16], and possibly associate with less side effects compared to antibiotics. Third, the production of phages is an inexpensive procedure that does not need sophisticated techniques. Finally, phages are associated with some other beneficial effects that could be of interest. These pleiotropic effects include immunomodulatory properties[24,25,26], inhibition of platelet adhesion to fibrinogen[27], and inhibition of tumor growth[28].

Several reports have demonstrated the efficacy of phages in the treatment of infectious diseases caused by Gram-negative bacteria, such as *Escherichia coli*, *Pseudomonas aeruginosa*, *Acinetobacter baumannii*, *Klebsiella pneumoniae*, *Vibrio vulnificus*, and *Salmonella* spp., and also Gram-positive bacteria, such as *Enterococcus faecium* and *Staphylococcus aureus*[7,8,9,10,11,12]. Given these characteristics, phages could be considered as potential antibacterial agents[29]. Moreover, phages could be employed as a powerful and fast tool for typing of bacterial strains such as those of *Klebsiella* spp., thereby being useful for diagnostic purposes[30].

In spite of the positive findings on the therapeutic efficacy of phages, this strategy has not been introduced into routine clinical practice for the treatment of bacterial infections. This stems from several reasons, the most important of which are the advent and widespread use of antibiotics in the Western world as well as the inconsistency and unsuccessful results of early trials. The main reasons for the inconsistent findings of the early trials are (1) inadequate scientific methodology that was used; (2) not heeding the prerequisites for phage therapy, such as lack of complete knowledge on phage biology, including lysogeny phenomenon (which might have led to the employment of a wrong phage); (3) lack of placebo control and robust trial design; (4) not identifying pure phage strains; (5) not meeting safety requirements for phage preparations, such as endotoxin removal; (6) not confirming adequate phage viability in the employed preparations; and (7) rapid clearance of phages from the body. The modern generation of phage research has attempted to overcome these shortcomings and promising results have been obtained. However, there is still much work to be done in order to extrapolate positive *in vitro* findings into more complicated *in vivo* experiments[31,32,33]. In addition, the development of phage resistance is a widespread phenomenon among bacterial hosts. This could occur through several mechanisms, such as mutation, blockade, or loss of phage receptors; prevention of phage DNA entry; lysogeny; acquiring restriction modification systems; and mutation of bacterial genes necessary for phage replication or assembly[33,34]. Although development of resistance mechanisms may coincide with loss of bacterial virulence, this is not generalizable to all cases and phage resistance is indeed among the main concerns of therapeutic application of phages[35].

In recent years, there have been relatively few studies on the efficacy of phage therapy against *Klebsiella* infection, particularly resistant *Klebsiella* spp. The promising results of this investigation add to the existing body of literature about the potential efficacy of phage therapy. As *Klebsiella* spp. are among the most important causes of noscomial infections[36], and their control has been faced with difficulty due to the spread of multidrug-resistant strains, further investigations in this field are greatly recommended.

As a limitation of the current study, it must be mentioned that the 24-h bacterial cultures were not tested for bacteriophage resistance. Furthermore, it would be helpful to evaluate the bactericidal efficacy of phage preparations in more detailed time points.

To sum, the results of this research support the idea that phages are effective bactericidal agents that could serve as potential alternatives for antibiotics in the treatment of resistant bacterial infections. In addition, the present findings provide evidence with respect to the impact of concentration, incubation duration, and method of culture on the bactericidal effects of phages.

## ACKNOWLEDGMENTS

This study was conducted with financial support that was provided by the Vice Chancellor for Research at the Mashhad University of Medical Sciences.
